# The Emergence of New Catalytic Abilities in an Endoxylanase from Family GH10 by Removing an Intrinsically Disordered Region

**DOI:** 10.3390/ijms23042315

**Published:** 2022-02-19

**Authors:** Carlos Gil-Durán, Romina V. Sepúlveda, Maximiliano Rojas, Víctor Castro-Fernández, Victoria Guixé, Inmaculada Vaca, Gloria Levicán, Fernando D. González-Nilo, María-Cristina Ravanal, Renato Chávez

**Affiliations:** 1 Facultad de Química y Biología, Universidad de Santiago de Chile (USACH), Santiago 9170022, Chile; cagild@gmail.com (C.G.-D.); gloria.levican@usach.cl (G.L.); 2 Center for Bioinformatics and Integrative Biology, Facultad de Ciencias Biológicas, Universidad Andrés Bello, Santiago 8370146, Chile; rosepulveda87@gmail.com (R.V.S.); maximiliano.rojas@unab.cl (M.R.); fernando.gonzalez@unab.cl (F.D.G.-N.); 3 Departamento de Biología, Facultad de Ciencias, Universidad de Chile, Santiago 7800003, Chile; vcasfe@uchile.cl (V.C.-F.); vguixe@uchile.cl (V.G.); 4 Departamento de Química, Facultad de Ciencias, Universidad de Chile, Santiago 7800003, Chile; inmavaca@uchile.cl; 5 Instituto de Ciencia y Tecnología de los Alimentos, Facultad de Ciencias Agrarias y Alimentarias, Universidad Austral de Chile (UACH), Valdivia 5090000, Chile

**Keywords:** GH10 endoxylanase, intrinsically disordered region, protein dynamics, (β/α)_8_-barrel domain, new activities

## Abstract

Endoxylanases belonging to family 10 of the glycoside hydrolases (GH10) are versatile in the use of different substrates. Thus, an understanding of the molecular mechanisms underlying substrate specificities could be very useful in the engineering of GH10 endoxylanases for biotechnological purposes. Herein, we analyzed XynA, an endoxylanase that contains a (β/α)_8_-barrel domain and an intrinsically disordered region (IDR) of 29 amino acids at its amino end. Enzyme activity assays revealed that the elimination of the IDR resulted in a mutant enzyme (XynAΔ29) in which two new activities emerged: the ability to release xylose from xylan, and the ability to hydrolyze *p*-nitrophenyl-β-d-xylopyranoside (pNPXyl), a substrate that wild-type enzyme cannot hydrolyze. Circular dichroism and tryptophan fluorescence quenching by acrylamide showed changes in secondary structure and increased flexibility of XynAΔ29. Molecular dynamics simulations revealed that the emergence of the pNPXyl-hydrolyzing activity correlated with a dynamic behavior not previously observed in GH10 endoxylanases: a hinge-bending motion of two symmetric regions within the (β/α)_8_-barrel domain, whose hinge point is the active cleft. The hinge-bending motion is more intense in XynAΔ29 than in XynA and promotes the formation of a wider active site that allows the accommodation and hydrolysis of pNPXyl. Our results open new avenues for the study of the relationship between IDRs, dynamics and activity of endoxylanases, and other enzymes containing (β/α)_8_-barrel domain.

## 1. Introduction

Xylan, one of the main components of plant hemicelluloses, is a polysaccharide composed of a backbone of β-d-xylose molecules linked by β-1,4 bonds, which can be substituted by different chemical groups [1]. Owing to its heterogeneity, xylan degradation requires the action of numerous enzymes. Among them, endoxylanases (endo-β-1,4-xylanases, E.C. 3.2.1.8) are key players, because they act on the backbone of xylan, hydrolyzing internal β-1,4 linkages between xylopyranosyl residues, giving rise to a diverse array of xilooligosaccharides (XOS) that may include xylobiose, xylotriose, xylotetraose, or longer, which may be or not branched by some substituents [1].

Based on amino acid sequence similarities, endoxylanases have been classified into different glycoside hydrolases (GH) families [2]. Family GH10 is one of the most known. Endoxylanases from this family have a classical (β/α)_8_-barrel fold [3,4]. Concerning activity, GH10 endoxylanases are versatile, acting on different kinds of xylans and XOS. This versatility is due to the presence of several subsites in the active site that accommodate the xylose moiety of different substrates [4,5]. GH10 endoxylanases hydrolyze glycosidic bonds by a double-displacement mechanism, with retention of anomeric configuration. Two glutamate residues separated by 5.5 Å are implicated in the catalytic mechanism of GH10 endoxylanases. One of these glutamates acts as the general acid/base, while the other one is the nucleophile [4,5,6].

Endoxylanases have many applications including animal feed, biofuel, baking, paper, and detergent industries [7]. In this regard, GH10 endoxylanases are of particular interest because of their versatility in the use of different substrates [4,5]. In recent years, some studies have shown that mutations in GH10 endoxylanases lead to alterations in substrate specificities of these enzymes [8,9]. A deep understanding of the molecular mechanisms underlying these changes could be very useful in the engineering of GH10 endoxylanases.

In previous work, we characterized XynA, an endoxylanase from the fungus *Cladosporium fildesense* belonging to the family GH10 [10]. XynA has a typical endo-*β*-1,4-xylanase activity, producing XOS as final products [10]. During the course of these experiments, we noticed that in addition to the typical (β/α)_8_-barrel fold found in GH10 endoxylanases, XynA contains an intrinsically disordered region (IDR) of 29 amino acids at its amino end (see Results). IDRs are protein regions that fail to form secondary or tertiary structures [11]. In nature, the majority of eukaryotic proteins contain IDRs [11], and these regions have been involved in several functions of proteins with impact on critical biological processes including cell signaling, cell cycle control, or diseases [12,13,14,15]. However, and despite the advances in our knowledge of IDRs, the mechanisms underlying the effects of these regions on enzyme catalysis still remain largely unknown.

In this work, we have eliminated the IDR in XynA. As a result, we obtained a mutant enzyme, named XynAΔ29, with increased flexibility, and in which two new activities emerged: the ability to release xylose from xylan, and the ability to hydrolyze *p*-nitrophenyl-β-d-xylopyranoside (pNPXyl), a substrate that wild-type enzyme cannot hydrolyze. It was of particular interest to determine why XynAΔ29 can hydrolyze pNPXyl. Molecular dynamics simulations revealed that the emergence of this new activity was correlated with a dynamic behavior not previously observed in GH10 enzymes: a hinge-bending motion of two symmetric regions within the (β/α)_8_-barrel domain, whose hinge point is the active cleft. The hinge-bending motion observed in XynAΔ29 is more intense than in XynA, and promotes the formation of a wider active site for the entry and accommodation of pNPXyl and its subsequent hydrolysis by catalytic residues. Our results open new avenues for the study of the relationship between IDRs, dynamics and activity of GH10 enzymes, and other enzymes containing (β/α)_8_-barrel fold.

## 2. Results

**Figure 1:**
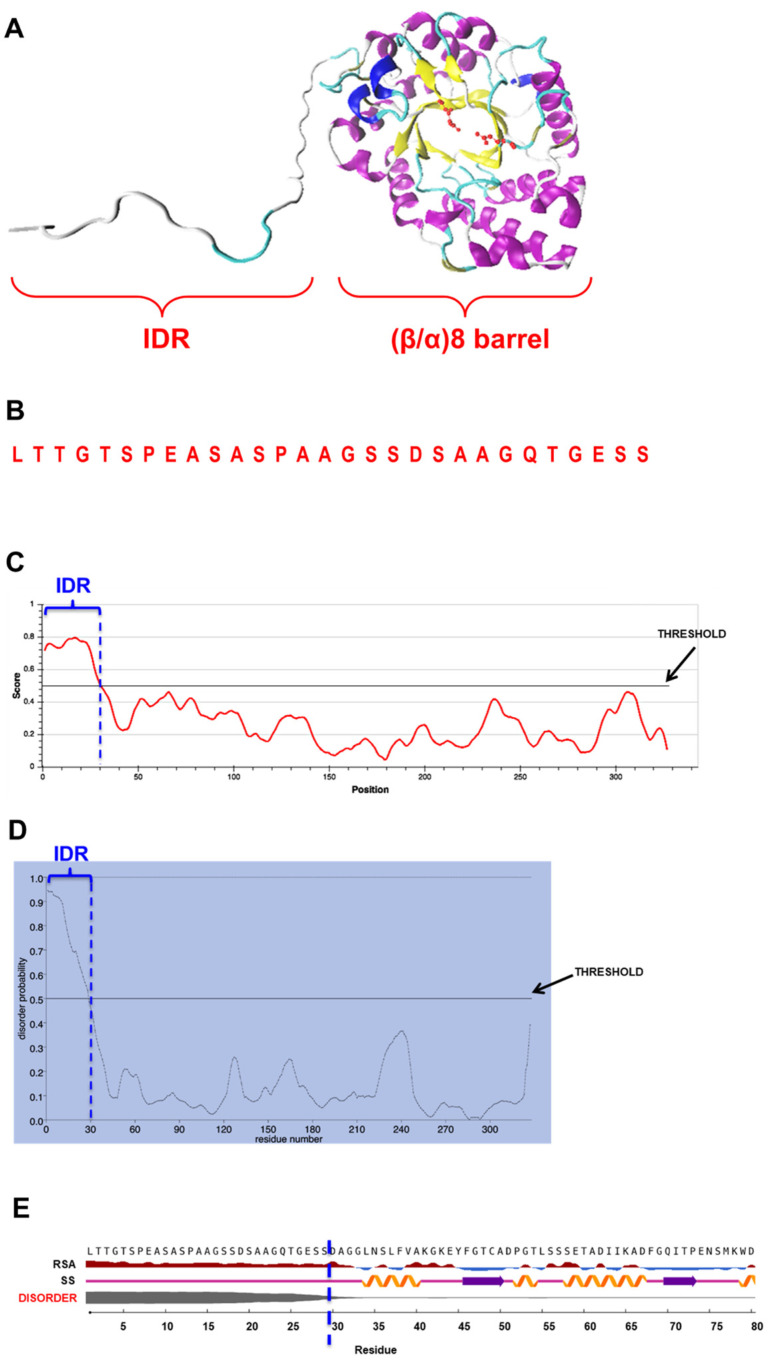
(**A**) Homology model of XynA, indicating the IDR and the (β/α)_8_-barrel fold; (**B**) Amino acid sequence of the IDR. (**C**–**E**) Outputs of the result of prediction of disordered regions in mature XynA by IUPred3 (**C**), PrDOS (**D**) and NetSurfP-2.0 (**E**). In all cases, the IDR of 29 amino acids appears delimited by a vertical dashed blue line. In (**C**,**D**), thresholds used to determine the disordered property by the respective programs are indicated. In (**E**), disorder property is indicated by a gray line, thicker line means higher disorder. Secondary structure (SS) predicted for IDR is coil structure (light violet line), while relative surface accessibility (RSA) predicts that this region is exposed (dark red).

As mentioned, we previously purified and characterized XynA, an endoxylanase from the fungus *Cladosporium fildesense* [10]. To gain insights into the molecular mechanisms of XynA, we analyzed the structure of the enzyme by bioinformatics tools. We generated a homology model for XynA (Figure 1A), and we noticed that in addition to the classical (β/α)_8_-barrel fold found in GH10 endoxylanases, XynA contains an intrinsically disordered region (IDR) of 29 amino acids at its amino end (Figure 1B), a region not found in other endoxylanases (Supplementary Figure S1).

The disordered property of the IDR was confirmed using three different bioinformatics tools, which are based on different methods for the detection of disordered regions in proteins: IUPred3 [16], NetSurfP-2.0 [17], and PrDOS [18]. The three bioinformatics tools predicted the disordered nature of this IDR (Figure 1C–E). This prediction was also supported by root mean square fluctuation (RMSF) analysis, which indicated that during 1-microsecond molecular dynamic simulations of XynA, the region comprising the IDR showed higher fluctuations as compared with the rest of the protein (Supplementary Figure S2). On the other hand, an estimation of evolutionary conservation of XynA was performed with ConSurf. No conservation between the IDR and the amino end of 149 similar proteins was detected (Supplementary Figure S3).

**Figure 2:**
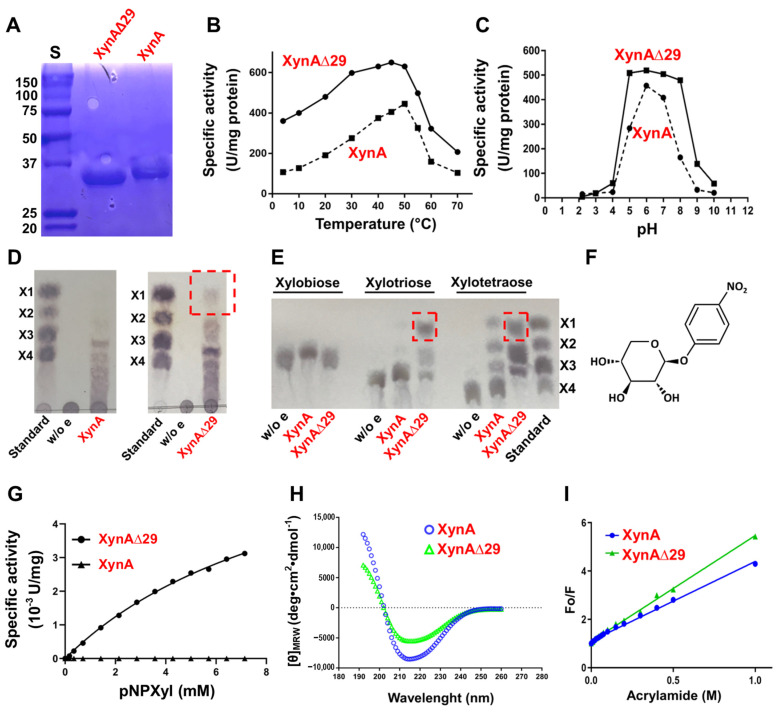
(**A**) SDS-PAGE of purified enzymes. S: Molecular weight standard in kDa. (**B**) Xylanolytic activity of XynA and XynAΔ29 at different temperatures. (**C**) Xylanolytic activity of XynA and XynAΔ29 at different pH. (**D**) TLC of the products released from xylan. (**E**) TLC of the products released from XOS. Xylose released by XynAΔ29 is highlighted in red boxes. Controls without enzymes (w/o e), and standards xylose (X1), xylobiose (X2), xylotriose (X3), and xylotetraose (X4) were included. (**F**) Structure of pNPXyl. (**G**) Michaelis–Menten plot of the hydrolysis of pNPXyl by XynA and XynAΔN29. (**H**) Far-UV circular dichroism spectra of XynA and XynAΔ29. The mean residue ellipticity ([θ]_MRW_) was plotted against wavelength. (**I**) Stern-Volmer plot for tryptophan fluorescence quenching by acrylamide of XynA and XynAΔ29. In (**B**,**C**,**G**,**I**) values are the mean of three experiments, bars representing the standard error of the mean lie within the size of the symbols.

To assess the impact of IDR in XynA function, we expressed and purified XynA and a mutant version of the enzyme named XynAΔ29, which lacks the IDR (Figure 2A), and we performed a comparative study of both enzymes. We observed that XynAΔ29 is more active than XynA in a wide range of temperature and pH values (Figure 2B,C). Concerning kinetic parameters, the enzymes have similar K_M_ (14.07 ± 2.3 mg/mL for XynA and 18.3 ± 1.0 mg/mL for XynAΔ29). However, the kcat values of XynAΔ29 (224.8 ± 6.2 seg^−1^) were about 1.7-fold higher than those observed for XynA (131.6 ± 10.7 seg^−1^), suggesting that XynAΔ29 has become more efficient in the hydrolysis of the substrate as compared to XynA.

Next, we addressed whether the elimination of IDR has an impact on products generated by enzymes. Interestingly, and different from the wild-type enzyme, XynAΔ29 acquired the ability to release xylose from polymeric xylan (Figure 2D). This ability also appears considerably increased when xylotriose and xylotetraose were used (Figure 2E). These results suggest that the elimination of IDR has modified the behavior of the enzyme, making XynAΔ29 able to generate xylose from xylan.

The improved ability of XynAΔ29 of releasing xylose from different substrates prompted us to explore whether this enzyme could have acquired β-xylosidase activity. β-xylosidases (EC 3.2.1.37) are enzymes that hydrolyze xylobiose and XOS to xylose from the non-reducing end [19]. To test the hypothesis mentioned, we used two β-xylosidase substrates: xylobiose (natural substrate of β-xylosidases), and *p*-nitrophenyl-β-D-xylopyranoside (pNPXyl, Figure 2F), a synthetic substrate widely used for measuring β-xylosidase activity. XynAΔ29 does not hydrolyze xylobiose (Figure 2E). However, and different from the wild-type enzyme, XynAΔ29 has the ability to hydrolyze pNPXyl, following Michaelis–Menten kinetics (Figure 2G) with Vmax = 5 ± 0.33 µM/min and K_M_ = 2.67 ± 0.44 mM. Taken together, the results described indicate that the elimination of IDR modified the classical endo-β-1,4-xylanase activity of XynAΔ29, resulting in the emergence of two new activities that wild-type enzyme does not show: a xylose-releasing 1,4-β-d-xylan xylohydrolase activity, and a pNPXyl-hydrolyzing activity.

Because XynAΔ29 can now hydrolyze pNPXyl (Figure 2G), it was of particular interest to explore how the elimination of a distal IDR at the amino end of the protein allows the hydrolysis of this new substrate. For this purpose, we performed biophysical experiments and molecular dynamics simulations. Far-UV circular dichroism revealed important changes in the secondary structure of XynAΔ29 as compared with XynA (Figure 2H). The percentage of secondary structure content determined from the CD spectra indicated that XynAΔ29 has a lower content of α-helix and parallel β-sheet, and increased content of short and irregular structures as compared to XynA (Supplementary Table S1). More interesting, these structural changes correlated with increased flexibility of XynAΔ29. As can be seen in Figure 2I, accessibility for tryptophan quenching by acrylamide in XynAΔ29 is enhanced as compared with XynA, which indicates that the mutant enzyme has a more flexible structure. This conclusion was also supported by molecular dynamics simulations, which show that tryptophan residues of XynAΔ29 have enhanced mobility as compared to XynA (Supplementary Videos S1 and S2), as well as thermal unfolding experiments, which indicate that XynAΔ29 has a slightly lower thermal stability than XynA (Supplementary Figure S4).

**Figure 3:**
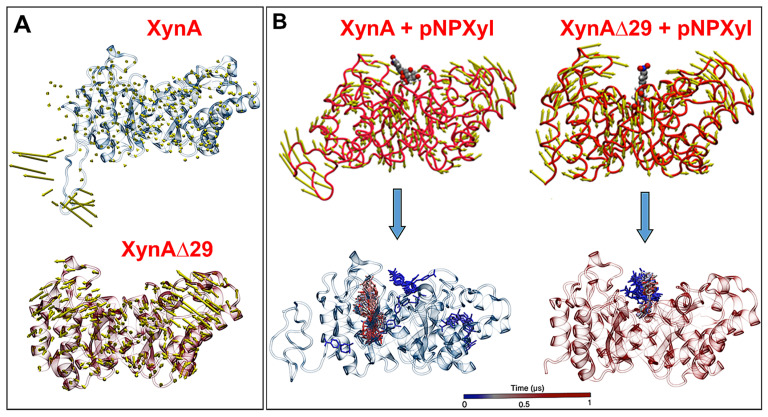
(**A**) Motion of XynA and XynAΔ29 described by normal mode vectors. The full dynamics can be seen in Supplementary Videos S3 and S4. (**B**) Interactions of pNPXyl with XynA and XynAΔ29, described by normal modes (upper figures), and tracking of pNPXyl movements in both enzymes during a trajectory of 1 µs (lower figures). Note in the lower figures that pNPXyl always correctly accommodates into the active cleft of XynAΔ29 while in XynA the substrate shows erratic positioning, mainly around the enzyme. The full dynamics can be seen in Supplementary Videos S5 and S6.

It is known that flexibility affects the intrinsic dynamics of proteins, thus being a key determinant in the substrate specificity of enzymes [20]. Therefore, we sought to determine whether the elimination of IDR in XynA changed the dynamics of the protein. We observed that in XynA, IDR shows large motion, whereas the core (β/α)_8_-barrel displays a quite rigid motion (Figure 3A, Supplementary Video S3). On the contrary, the (β/α)_8_-barrel domain of XynAΔ29, now released from IDR, acquired a more dynamic motion, as depicted by increased normal mode vectors (Figure 3A, Supplementary Video S4).

**Figure 4:**
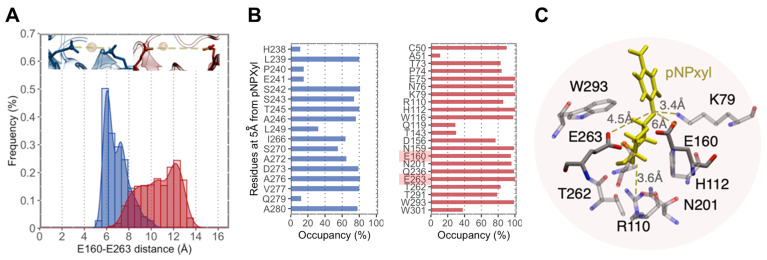
(**A**) Distribution of distances between E160 and E263 in XynA (blue) and XynAΔ29 (red) measured during a trajectory of 1 µs. (**B**) Frequency of residue occupancy of XynA (blue) and XynAΔ29 (red) by pNPXyl during the same trajectory. In XynA the substrate visits residues outside the active site, while in XynAΔ29 the substrate interacts with amino acids of the active site, including E160 and E263 (highlighted). (**C**) Interactions of pNPXyl (yellow) with relevant amino acids of the active site of XynAΔ29, including catalytic residues E160 and E263.

Molecular docking experiments showed that the increased motion displayed by the (β/α)_8_-barrel of XynAΔ29 is critical to explain why the mutant enzyme can hydrolyze pNPXyl. In both enzymes, the presence of pNPXyl induces an interesting dynamic behavior: a hinge-bending motion of two symmetric regions within the (β/α)_8_-barrel, whose hinge point is the active cleft (Figure 3B). However, in XynA, the presence of pNPXyl yields “unproductive” interactions because during a trajectory of 1 microsecond, pNPXyl mainly moves around the enzyme and the vicinity of active cleft, but is not stably retained into the active site (Figure 3B, Supplementary Video S5). On the contrary, the hinge-bending motion of the (β/α)_8_-barrel is clearly more increased during the interaction between XynAΔ29 and pNPXyl, allowing the entry of substrate to the active cleft of XynAΔ29, and its stable accommodation there during the complete 1 µs trajectory (Figure 3B, Supplementary Video S6). The comparison of active clefts of XynA and XynAΔ29 along their trajectories suggest that the increased hinge-bending motion in the (β/α)_8_-barrel of XynAΔ29 promotes the formation of a wider active site for the entry of pNPXyl, including an increase of the distance between the catalytic residues E160 and E263. Thus, while the maximal distance observed between E160 and E263 in XynA is 6.0 Å, the dynamics acquired by XynAΔ29 create a pocket where E160 and E263 display a longer distance that reaches a peak of 12 Å (Figure 4A). Different from XynA, this wider active site allows the accommodation of the glycosyl group of pNPXyl in the active site of XynAΔ29, its stable interaction with amino acids located there, and the hydrolysis of the glycosidic linkage by catalytic residues E160 and E263 (Figure 4B,C).

## 3. Discussion

Previous studies have shown that the amino end region is important for the stability and activity of endoxylanases [21,22,23,24,25,26,27,28,29,30]. In some cases, these amino ends have been described as “non-structured”, or contain amino acids predicted to be disordered residues [22,23,24]. Two of these cases correspond to endoxylanases from family GH11 [23,24], while only one corresponds to an endoxylanase from family GH10 [22]. This single case is the endoxylanase Xyn10 from *Aspergillus niger*, where five disordered residues were eliminated from the amino end. Among other effects, the mutant enzyme showed a decrease in the optimum temperature for the activity of 6 °C, and an increase of K_M_, whereas no changes were observed in optimum pH [22]. These results partially differ from our results, because we do not find differences in K_M_; however, we observed important changes in activity at different pHs (Figure 2C). At this point, it is important to mention that our results (Supplementary Figures S1 and S3) and previous sequence similarity studies [21] have shown that the amino end of GH10 endoxylanases has a low degree of conservation, which suggests that they do not necessarily have similar roles in endoxylanases.

Although there are some natural GH10 endoxylanases able to release xylose from xylan [31,32], in general terms GH10 endoxylanases do not show this property. In the case of XynA, a previous work [10] and the result shown in Figure 2D confirmed that this endoxylanase lacks this ability. However, after the elimination of the IDR, the resulting enzyme XynAΔ29 acquired the ability to release xylose from xylan (Figure 2D). As far as we know, XynAΔ29 represents the first case of an endoxylanase acquiring the ability to release xylose from xylan after the elimination of its amino end. Concerning the activity towards xylooligosaccharides, XynAΔ29 has no activity on xylobiose, but it shows an increase in activity on xylotriose and xylotetraose with respect to XynA. Similar results on xylotriose have been reported in other fungal endoxylanases where their respective amino ends were deleted [27,28,29].

As mentioned, XynAΔ29 cannot hydrolyze xylobiose, discarding that the enzyme had acquired β-xylosidase activity. However, XynAΔ29 shows activity on the artificial substrate pNPXyl, which is commonly used to measure β-xylosidase activity. Although there are some endoxylanases that have the natural ability to hydrolyze pNPXyl [33,34], XynAΔ29 represents the first case of an endoxylanase acquiring such ability in vitro after the elimination of an IDR. While the kinetic parameters observed for the hydrolysis of pNPXyl by XynAΔ29 are modest as compared with true β-xylosidases, this result represents an interesting starting point for the future evolvement of XynAΔ29, aiming for the obtainment of a bifunctional endoxylanase/β-xylosidase. Endoxylanases and β-xylosidases (and other glycoside hydrolases) are believed to proceed from a common ancestor [35,36], so the eventual obtaining of this evolved endoxylanase/β-xylosidase would be an important verification of this hypothesis.

Regarding the accommodation and hydrolysis of pNPXyl by XynAΔ29, our in silico analyses support the experimental results. The active site of GH10 endoxylanases has several subsites, named −3, −2, −1, +1, +2, and +3 subsites. The two catalytic glutamic acids hydrolyze the substrate between -1 and +1 subsites. The −3, −2, −1 subsites (named glycone subsites) are well conserved and allow the accommodation of the part of the molecule that contains the non-reducing end [3,6,37,38]. On the other hand, the +1, +2, and +3 subsites (named aglycone subsites) are less conserved and accommodate the part of the molecule corresponding to the leaving group of the hydrolysis [3,6,37,38]. The frequency of residue occupancy shows that pNPXyl interacts with residues K79, H112, W116, N159, Q236, W293, and W301 in XynAΔ29 (Figure 4B), which are conserved amino acids found in the subsite −1 of GH10 endoxylanases [5]. More importantly, the molecular simulation experiments showed that pNPXyl docks in the expected position in the active site of XynAΔ29, that is, the xylose moiety in the subsite -1, the *p*-nitrophenyl moiety (aglycone) in subsite +1, and the catalytic residues E160 and E263 between both subsites, hydrolyzing the glycosidic bond (Figure 4C and Supplementary Video S6).

It has been described that IDRs can influence protein dynamics by different modes of action [11]. In our case, we observed that the elimination of IDR in XynA increases flexibility and motion of the (β/α)_8_-barrel domain. Dynamics is regarded as a key factor in the function of enzymes [39,40,41], and it is intimately related to protein flexibility [11,42]. Our results support this notion suggesting that relationships between IDR, flexibility, and dynamics determine the marked differences in the activity observed in XynA and XynAΔ29. At this point, it should be mentioned that it is not possible to predict which of the 29 amino acids are most important for the function of the IDR. Although about 20 amino acids have a clear propensity for disorder (Figure 1), the quantitative evaluation performed with ConSurf (Supplementary Figure S3) showed no conservation between the IDR and the amino end of 149 similar proteins. Therefore, in the future, an extensive mutagenesis campaign will be needed to determine the importance of each amino acid in IDR function. Such an analysis is far beyond the scope of the present investigation.

As far as we know, the hinge-bending motion observed in XynA and XynAΔ29 has not been previously described in other endoxylanases from family GH10. However, this kind of motion is usual in endoxylanases from family GH11. The catalytic domain of GH11 endoxylanases has a β-jelly roll structure that resembles a partially closed hand, where the access of the substrate to the active cleft is controlled by hinge-bending motion between “thumb” and “fingers” regions [43]. It has been observed that mutations in key residues of the hinge in GH11 endoxylanases lead to an increase in catalytic efficiency that may be due to improved flexibility of the enzyme [44]. Moreover, it has been suggested that changes in motion and flexibility of the hinge region in GH11 endoxylanases, after the substrate binding, could produce changes in the accessibility of the active site [45]. In our case, molecular docking experiments showed that the increased motion displayed by the (β/α)_8_-barrel of XynAΔ29 in the presence of pNPXyl promotes the formation of a wider active site for the entry of this substrate allowing its accommodation and hydrolysis by the catalytic residues. Taken together, these results suggest that similarly to GH11 endoxylanases, the hinge-bending motion, associated with changes in flexibility, is a mechanism that also can modify the access, binding, and hydrolysis of substrates by GH10 endoxylanases, even allowing the use of substrates not normally used by these enzymes such as pNPXyl.

Overall, our results suggest that XynAΔ29 is no longer a typical endoxylanase like XynA, but rather should be considered as a bifunctional 1,4-β-d-xylan xylohydrolase /pNPxyl hydrolase. The term bifunctionality refers to the ability of an enzyme to possess two different catalytic activities on the same polypeptide chain [46]. Bifunctional endoxylanases have great biotechnological potential because their characteristics allow more efficient xylan hydrolysis, thereby expanding its possible applications at an industrial level. In this context, the improvement of activity observed in XynAΔ29, as well as the eventual directed evolution of XynAΔ29 to a bifunctional endoxylanase/β-xylosidase mentioned before, would be of great impact for biotechnological purposes.

As a concluding remark, it is important to mention that around 10% of enzymes in nature contain (β/α)_8_-barrel structures [47]. Therefore, the results obtained in this work are not only interesting for the study of the relationship between IDR, dynamics, and catalytic properties in other endoxylanases from family GH10, but also suggest that dynamics and activities of other enzymes with (β/α)_8_-barrel structures could also be modified by the presence/absence of IDRs.

## 4. Materials and Methods

### 4.1. Prediction of Disordered Nature of IDR

The deduced sequence of XynA (GenBank accession MG007677), lacking signal peptide, was used as input for the prediction of disordered regions. For this purpose, three different bioinformatics tools for the prediction of protein disorder, based on different methods, were used: IUPred3 (https://iupred3.elte.hu/) (accessed on 18 August 2021), NetSurfP-2.0 (https://services.healthtech.dtu.dk/service.php?NetSurfP-2.0) (accessed on 18 August 2021), and PrDOS (https://prdos.hgc.jp/cgi-bin/top.cgi) (accessed on 18 August 2021). In all cases, predictions were performed using default parameters.

IUPred3 predicts the tendency for each amino to be in a disordered region, as well as context-dependent disordered regions. The algorithm uses an empirical force field to estimate the energy for each residue based on its interactions with other residues in the structure [16]. The output consists of a graphical prediction of the probability of disorder of each residue in the protein. The threshold score set to determine the disordered property is 0.5.

NetSurfP-2.0 predicts disordered regions in a sequence by using convolutional and long short-term memory neural networks trained on solved protein structures [17]. As a result, NetSurfP-2.0 gives the probability of disorder of the residues, secondary structure, and relative surface accessibility of the IDR.

PrDOS uses two complementary algorithms. One algorithm works on the basis of local amino acid sequence, specifically using a position-specific score matrix of the sequence to map individual residues in a given sliding. The second algorithm compares the input sequence with homologous proteins for which structural information is available. The final prediction combines the results of the two algorithms. The prediction includes a predicted disorder probability of each residue, with a default false positive rate set at 5% [18].

Finally, the degree of evolutionary conservation of XynA was estimated with ConSurf, using default parameters (https://consurf.tau.ac.il/) [48] (accessed on 3 January 2022).

### 4.2. Expression of XynA and XynAΔ29 in Pichia Pastoris, and Purification of Enzymes

The complete experimental detail for expressing XynA in *Pichia pastoris*, and its further purification, was previously described [10].

For the expression and purification of XynAΔ29, the same protocol was followed, with some variations. The *xynA* cDNA from *Cladosporium fildesense* [10] was used as a template to obtain the coding sequence of XynAΔ29 by PCR. This sequence was amplified using primers Picz-xylΔ29N-EcorI-fw (5′-AGACTCCGAATTCGACGCTGGAGGCCTCAAC-3) and Picz-Xyl-SacII-Rv (5′-AGACTCCCGCGGCTGCGAGGAGGGTGGTGAG-3). To avoid undesired mutations, *Pfu* DNA polymerase (Invitrogen, Carlsbad, CA, USA) was used and the amplicon was sequenced previously to use. The amplicon obtained was digested with *Eco*RI and *Sac*II, and cloned into pPICZα-A, thus giving rise to plasmid pPICZαA-XynAΔ29. This plasmid was used to transform electrocompetent *P. pastoris* GS115 cells. Transformed clones were selected on zeocin (100 mg/mL). Five zeocin-resistant clones were selected, and grown in liquid media according to the instructions of the Easy-Select Pichia Expression kit (Invitrogen, Carlsbad, CA, USA). Xylanase expression was inducted by adding methanol daily (1% final concentration), over 4 days. Aliquots were withdrawn daily for measurement of xylanolytic activity (see below).

All clones showed similar xylanolytic activity, so one of them was randomly chosen for purification of XynAΔ29. The selected transformant was grown in a liquid medium (2 L) over 4 days and induced with methanol as described above. After induction, the culture supernatant was separated and concentrated to a final volume of 10 mL. Purification of XynAΔ29 was performed by affinity chromatography using His Pure Ni-NTA resin (Thermo Fisher Scientific, Waltham, MA, USA). The resin was regenerated in 50 mL of 50 mM sodium phosphate buffer pH 8.0. A 1.6 × 10 cm column was loaded with 5 mL of the resin bed and equilibrated with 50 mL of the same buffer, with a flow rate of 10 mL/hour. Then, the column was cooled at 4 °C and washed for 3 h with a 50 mM sodium phosphate buffer plus 0.5 M NaCl. Finally, the column was loaded with 4 mL of the protein concentrate and washed with three volumes of the same buffer at a rate of 10 mL/hour. The column was eluted with a step gradient from 0 to 200 mM imidazole in 50 mM sodium phosphate buffer pH 8.0. Fractions of 4 mL were collected, pooled, and analyzed for xylanolytic activity (see below). Purity was estimated by SDS-PAGE stained with Coomassie brilliant blue R-250.

### 4.3. Measurement of Xylanolytic Activity

For the measurement of xylanolytic activity, reducing sugars were detected by the classical 3,5-dinitrosalicylic acid (DNS) assay [49]. Briefly, 450 µL of a 1% beechwood xylan (Carl Roth, Karlsruhe, Germany) solution was pre-incubated in 50 mM citrate buffer pH 5.3 for 10 min at 50 °C. Then, 50 µL of the sample was added, and the mix was incubated for an additional 10 min at 50 °C. The reaction was stopped with 750 µL of DNS reagent (1% 3,5-dinitrosalicylic acid, 30% sodium potassium tartrate, 1.6% NaOH), and incubated at 100 °C for 10 min. The samples were cooled to room temperature and centrifuged to recover supernatant and remove residual xylan. Absorbance at 540 nm was measured in the supernatant using a spectrophotometer. For the calibration curve, pure xylose was used in concentrations ranging from 0 to 20 mM. One unit (U) of enzyme activity was defined as the amount of enzyme necessary to produce 1 µmol of reducing sugars per minute.

### 4.4. Effect of pH and Temperature on Enzyme Activity

The effect of pH on XynA and XynAΔ29 activities was carried out in a pH range between 2.2–10. For the range 2.2–8.0, 100 mM McIlvaine buffers at different pH were prepared by using different amounts of 0.1 M citric acid and 0.2 M basic disodium phosphate stock solutions. For the range 9–10, 50 mM glycine-NaOH buffers were used. The reaction mix contained 20 µg (50 µL) of enzyme and 450 µL of 1% beechwood xylan solution. Each reaction was incubated for 10 min at 50 °C and, afterwards, the enzyme activity was measured as described above.

To determine the effect of temperature on XynA and XynAΔ29 activities, the reaction mix contained 20 μg of enzyme (50 μL) and 450 μL of 1% beechwood xylan solution, prepared at pH 6.0 in McIlvaine buffer. The reactions were incubated at different temperatures for 10 min. Then, xylanolytic activity was measured as described before.

In all cases, assays were performed in triplicate, and a standard deviation of the three independent experiments was calculated.

### 4.5. Thin Layer Chromatography (TLC) Experiments

Xylan was prepared at 1% in 50 mM citrate buffer pH 6.0. A total of 450 μL of xylan solution was incubated with 50 μL of pure XynA or XynAΔ29 (0.4 mg/mL) and incubated at 50 °C for 10 min. After incubations, TLC experiments were performed using silica gel 60G F254 plates (Merck, Darmstadt, Germany). Plates were activated at 100 °C for 30 min. After cooling the plate, 10 μL of each reaction were loaded. The mobile phase used was ethyl acetate: glacial acetic acid: water 3:2:1 (vol/vol/vol). After the plate was dried, spots were visualized by spraying a mixture of 0.2% orcinol and 10% sulfuric acid in ethanol, and heating at 85 °C for 10 min.

For TLC experiments with xylobiose, xylotriose and xylotetraose, they were prepared at 10 mg/mL in 50 mM citrate buffer pH 6.0. Reactions contained 4 μL of each XOS solution and 4 μL of each pure enzyme and were incubated at 50 °C for 10 min. Chromatography was performed exactly as was described above.

### 4.6. Determination of Kinetic Parameters for p-Nitrophenyl-β-d-xylopyranoside (pNPXyl)

For the determination of K_M_ and Vmax using pNPXyl, solutions with different concentrations of the substrate (between 0 and 10 mM) were used, with increases in the concentration of 1 mM. Each reaction was carried out using 50 µL of each pNPXyl solution and 20 µL of each pure enzyme (0.4 mg/mL). Each reaction was incubated for 10 min at 40 °C and stopped by adding 50 μL of 0.2 M sodium carbonate. Then, absorbance was measured at 405 nm. A calibration curve using pure *p*-nitrophenol (pNP) was prepared and used to determine enzymatic activity. One unit of enzyme activity (U) was defined as the amount of enzyme necessary to release 1 µmol of pNP per minute of the assay. The kinetic parameters were determined by non-linear regression of the activity values obtained, using the Michaelis–Menten equation. For this purpose, the GraphPad Prism version 7.0 program was used.

### 4.7. Determination of Protein Flexibility by Acrylamide Fluorescence Quenching

The conformational flexibility of XynA and XynAΔ29 was analyzed through tryptophane accessibility by acrylamide fluorescence quenching experiments. Experiments were carried out in a Jasco FP-8300 spectrofluorometer (Jasco Inc., Easton, PA, USA) at 25 °C. The samples at concentration 5 μM were prepared in 100 mM McIlvaine buffer pH 6.0 (see above) and excited at a wavelength of 295 nm. The emission spectra were recorded in the range of 305 and 450 nm, with a bandwidth of 5 nm. Each emission spectrum was corrected by subtracting the spectrum from the protein-free buffer (blank). For quenching, small aliquots of a 5 M acrylamide solution were added to the samples to obtain final concentrations ranging from 5 to 1000 mM acrylamide. The area under the curve (AUC) of spectra was calculated and fitted to the Stern–Volmer equation: F_0_/F = 1 + K_SV_ [Q] where F_0_ is the AUC fluorescence in the absence of quencher, F corresponds to the AUC fluorescence at a given quencher concentration, K_SV_ is the Stern–Volmer constant, and [Q] is the quencher (acrylamide) concentration.

### 4.8. Circular Dichroism Measurements

Far ultraviolet circular dichroism spectra were recorded in a Jasco J-1500 spectropolarimeter (Jasco Inc., Easton, PA, USA) using 0.1 cm path length quartz cells. Each spectrum is an accumulation of 3 scans measured in a continuous mode, with 2 s of digital integration time and a speed of 50 nm/min. Spectra were recorded between 192–260 nm at 25 °C. Protein concentrations were 0.151 mg/mL for XynA and 0.102 mg/mL for XynAΔ29, both prepared in 10 mM McIlvaine buffer pH 6.0. For the estimation of secondary structure content of proteins, the CD spectra were analyzed using the Beta Structure Selection method (BeStSel; http://bestsel.elte.hu/index.php) [50] (accessed on 9 January 2022).

For the analysis of the melting temperature of the enzymes, the heat-induced unfolding spectra were performed following the ellipticity at 220 nm with a temperature ramp of 3 °C/min. A Peltier system PTC-517 (Jasco Inc., Easton, PA, USA) was coupled to the spectropolarimeter for temperature control.

### 4.9. Homology Modeling and Molecular Dynamics Simulations

Homology models for XynA and XynAΔ29 were constructed with Modeller 9.10, using the structure of the endo-*β*-1,4-xylanase from *Aspergillus niger* (Protein Data Bank id: 4XUY) as a template. This protein has 59% identity with XynA. Models were refined and implanted in a water box of TIP3P molecules neutralized at 0.15 M NaCl. On the other hand, the pNPXyl molecule was parameterized using LigPrep [51].

For molecular dynamic simulations, four molecular systems representing the combinations of XynA and XynAΔ29 with or without pNPXyl were arranged. The arrangement of systems followed the recommended protocol by Schrödinger [52]. The first stage consisted of 20,000 steps of energy minimization and 40 ns of equilibration, framed in an NPT ensemble at 310.15 K and 1 atm. In addition, to preserve the secondary structure of the model, gradually decreasing harmonic restraints from 10 to 0.1 kcal/(mol · Å^2^) were applied to the backbone of the protein.

All molecular dynamics simulations were performed with the AMBERv18 software using the ff14SB, GLYCAM, gaff, and lipid14 force fields. Bonded and short- and long-range non-bonded interactions were integrated with a time step of 2. A 10-Å spherical cutoff was used for short-range non-bonded interactions. Each final trajectory reached 1 microsecond. All the four molecular dynamics simulations were very stable, with root mean square deviation (RMSD) values fluctuating around 1.0–1.8 Å throughout the 1 microsecond simulation period (Supplementary Figure S5).

## Data Availability

All data are included in the article and/or supporting information.

## Supplementary Materials

The following supporting information can be downloaded at: https://www.mdpi.com/article/10.3390/ijms23042315/s1.
