# Supplementary material for: The Emergence of New Catalytic Abilities in an Endoxylanase from Family GH10 by Removing an Intrinsically Disordered Region
Source: Int J Mol Sci. 2022 Feb 19;23(4):2315. doi: 10.3390/ijms23042315 (PMC8874783; doi:10.3390/ijms23042315)
Supplement: Supplementary file 1 [file ijms-23-02315-s001.zip › ijms-1542757-Supplementary figures and table.pdf]

```
KAH8651972.1            -------------------AAVPVIDERAIASIDDLFVAKGKTYYGTITDPTLLSNSQN     40 KAH7142868.1            --------
------------AAVPLVEERAVANIDDLFVAKGKKYYGTIADPALLSNSQN      40 CRG90418.1           -----------------------
SSNSSQLASINQLFQAKGKNYFGSITDPVDIANTQD       36 KFY28480.1             ----------------------
ATPVKRELASIHELFTAKGKLYYGSITDPVDIANQQD      37 XP_007712311.1          --------------
ASPFAEPEAFLEERQAAQSLDAAMKAKGRKYFGTATDPGRFNQGKN  46 XP_007697074.1           --------------
ASPFAEPEAFLEERQAAQSLDAAMKAKGRKYFGTATDPGRFNQGKN  46 XP_007687276.1           --------------
ASPFAEPEAFLEERQAAQSLDAAMKAKGRKYFGTATDPGRFNQGKN  46 XynA
LTTGTSPEASASPAAGSSDSAAGQTGESSDAGGLNSLFVAKGKEYFGTCADPGTLSSSET            60 RMY20991.1            ----------
--------ALPSAHEARQASAGSIDAAFKAKGKKYWGTIADRGTLSNTQN     42 OLN81304.1           ---------------------
AAPLEERQASQSIDRLFKAKGKQYFGTATDKGVLSSGKT                  39 BAA92882.1           -------------------------
SQQAPASINNAFVTKGKKYFGTCADQGTLSDGTN         34 Q96VB6.1             --------------------------
SQQAPASINNAFVAKGKKYFGTCADQGTLSDGTN         34 KAF4307014.1           ----------------------
GGVLPRQAAESINDKFVAKGKKYFGTCTDQGLLSSGSS38 KAH7045322.1           ----------------------
GAVLPRQAAESINEKFVAKGKKYFGTCADQGTLTSGSN38
                                  .:.  : :**: *:*: :*    : .


KAH8651972.1            AAVIKGSFGQLTCENSMKWDATEGTRGQFTFTNADTVVNFAQSNGILMRGHTLLWHSQLP            100
KAH7142868.1            AAVIKGSFGQLTCENSMKWDATEGTRGQFTFTNADTVVKFAQSNGILMRGHTLLWHSQLP            100
CRG90418.1             ANTIIADFGAVTPENSMKWDATEPSRGTFDFDGADQFVEFATSNDLIIRGHNLLWHSQLP   96 KFY28480.1
VDTIKAYFGQLSPQNSMKWDATESARGEFNFDGSDQFVDFATSNGMLIRGHTLLWHSQLP          97 XP_007712311.1
AAIIKANFGQITPENSMKWDATESTRGKFTFGTADQTAKFAKDNGKLIRGHTTIWHSQLP          106 XP_007697074.1
AAIIKANFGQITPENSMKWDATESTRGKFTFGTADQTAKFAKDNGKLIRGHTTIWHSQLP          106 XP_007687276.1
AAIIKANFGQITPENSMKWDATESTRGKFTFGTADQTAKFAKDNGKLIRGHTTIWHSQLP          106 XynA
ADIIKADFGQITPENSMKWDALEATRGVFTFDTADQTADFAAENGKMLRCHTLLWHSQLP          120 RMY20991.1
TNIIKADFGQLTAENSWKWDAIEPSRGSFSFGGADQLANFATSNGKILRGHTLLWHSQLP          102 OLN81304.1
ADVIKADFGQVTPENSMKWDATEPQRGQFIFGNSDYLVNFAQQNGKLVRGHTLLWHSQLP          99 BAA92882.1
SGIIKADFGQLTPENSMKWDATEPSQGKFSFSGADYLVNYAATNNKLIRGHTLVWHSQLP          94 Q96VB6.1
SGIIKADFGQLTPENSMKWDATEPSQGKFSFSGADYLVNYAATNNKLIRGHTLVWHSQLP          94 KAF4307014.1
AAVIKADFGQVTPENSMKWDATEPSQGSFSFDGADYLVDWATSNDKLIRGHTTVWHSQLP          98 KAH7045322.1
AAIIKADFGQVTPENSMKWDAVEPSQGNFNFAGADYLVDFATTNNKLIRGHTTVWHSQLP          98
                     * . ** :: :** **** *  :* * *  :*   ..:*  *. ::* *. :******


KAH8651972.1            SWVMSITNTAELKSVIENHITTVVKHFKGKIYAWDVVNEIFAEDGSLRDSVFSRVLGEEF    160 KAH7142868.1
SWVSSITNAAELKSVIENHVTTVVKHFKGKIYAWDVVNEIFAEDGSLRVSVFSKVLGEEF          160 CRG90418.1
SWVSDISDPDDLTEVIQNHISTVAGRYAGKIYAWDVVNEIFDEDGSLRDSVFSRVLGDQF          156 KFY28480.1
TWVSDISDPAELTEVIETHVSTVAARYAGKIYAWDVVNEIFAEDGTLRDSVFTRVLGDEF          157 XP_007712311.1
SWVSSIKDKATLTTVMQNHISSVMGHFKGQIYAWDVINEMFEENGNFRASVFYNVLGEDF          166 XP_007697074.1
GWVSSIRDKATLTTVMQNHISSVMGHFKGQIYAWDVINEMFEENGSFRASVFYNVLGEDF          166 XP_007687276.1
SWVSSIRDKATLTTVMQNHISSVMGHFKGQIYAWDVINEMFEENGNFRASVFYNVLGEDF          166 XynA
TWVEGITDKAELTEVIENHIAEVAGHFKGKCFAWDVVNEIFAEDGTLRDSVFSRVLGEDF          180 RMY20991.1
QWVQNINDRNTLTTVIQNHIAGVMGHYKNKVYSYDVVNEIFAEDGTLRDSVFSRVLGEDF          162 OLN81304.1
AWVKNVRDKATLTKVIEDHIAAVAGRYKGKIHHWDVVNEIFNENGSLRDSVFSEVLGEDF          159 BAA92882.1
SWVQGITDKNTLTSVLKNHITTVMNRYKGKVYAWDVVNEIFNEDGTLRSSVFYKVLGEDF          154 Q96VB6.1
SWVQGITDKNTLTSVLKNHITTVMNRYKGKVYAWDVVNEIFNEDGTLRSSVFYNVLGEDF          154 KAF4307014.1
SWVSSITDKDTLTSVIQNHVSTEIGRYKGKIYAWDVVNEIFNEDGSLRSSVFYNVLGEDF          158 KAH7045322.1
SWVSSITDKSTLTKVIQDHVSKEIGRYKGKIYAWDVVNEIFNEDGTLRSSVFYNVLGEDF          158
                    ** .: :   *. *:: *::    :: .: . :**:**:* *:*.:* *** .***.:*


KAH8651972.1            VSIAFKAARAADPNAKLYINDYNLDSATYGKVANGIVPKVKKWIAAGVPIDGIGSQSHLS   220 KAH7142868.1
VSIAFKAARAADPNAKLYINDYNLDSATYGKVANGIVPKVKKWIAAGVPIDGIGSQSHLS          220 CRG90418.1
VSIAFNAARKADPNAKLYINDYNLDSASYAKLANGIVPHVKEWIAAGVPIDGIGSQSHLQ          216 KFY28480.1
VSIAFNAARKADPDAKLYINDYNLDSAEYGKVANGMVPHVKEWVAAGVPIDGIGTQSHLG          217 XP_007712311.1
VRIAFEAAKKADPTAKRYINDYNLDTANYAKT-QAMAKNVKKWIAAGIPIDGIGSQTHLT          225 XP_007697074.1
VRIAFEAAKKADPSAKRYINDYNLDTANYAKT-QALAKNVKKWIAAGVPIDGVGSQTHLT          225 XP_007687276.1
VRIAFEAAKNADPAAKRYINDYNLDTANYAKT-QAIAKNVKKWIAAGVPIDGIGSQTHLT          225 XynA
VSIAFEAAKKAAPDAKRYINDYNLDSATYPKL-TGLAAKVKEWIAAGVPIDGIGSQSHLS          239 RMY20991.1
VRIAFEAARKADPNAKLYINDYNLDVASYAKL-TGLVNKVKQWRAAGIPIDGIGSQSHLS          221 OLN81304.1
VGIAFRAARKADPNAKLYINDYNLDTATYAKT-QGMAKKVKEWIGKGIPIDGIGSQTHLG          218 BAA92882.1
VRIAFEAARAADPQAKLYINDYNLDSANYGKT-TGLANHVKKWIAQGIPIDGIGSQTHLS          213 Q96VB6.1
VRIAFETARAADPQAKLYINDYNLDSANYGKT-TGLANHVKKWIAQGIPIDGIGSQTHLS          213 KAF4307014.1
VRIAFEAARAADPDAKLYINDYNLDSATYAKT-TGLISKVKEWIAAGVPIDGVGSQSHLS          217 KAH7045322.1
VRIAFEAARAADPNAKLYINDYNLDSATYAKT-TGLISKVKQWIAAGVPIDGIGSQSHLS217
                    * ***.:*: * * ** ******** * * *    .:  :**:*  . *:****:*:*:**


KAH8651972.1            AGG---SSGTQAALQALASSGVSEVAVTELDIANAPSADYVAVTKACLNVPKCVGITVWG  277 KAH7142868.1
AGG---SGGTQAALQALANSGVSEVAVTELDIANAPSADYVAVTKACLNVPKCVGITVWG          277 CRG90418.1            EGG-
--SSGTLGALRAMS-AVVSEVAITELDIANAPPDDYVAVTSACLQVENCVGITVWG        272 KFY28480.1           EGG---
SSGTLAALQAMS-AVVSEVAITELDIANAPAADYVAVTDACLQVANCVGITTWG         273 XP_007712311.1          AGQ---
GAATIDAMKLLC-SVASECAMTEVDIQNAQQADWTNVTKACLNQKNCVGITVWG         281 XP_007697074.1          SGQ---
GAATIDAMKLLC-SVASECAMTEVDIQNAQQADWTNVTKACLNQKNCVGITVWG         281 XP_007687276.1          SGQ---
GAATIDAMKLLC-SAASECAMTEVDIQNAQQADWTNVTKACLNQKNCVGITVWG         281 XynA           ES-----SGTADALKALA-
AAAPEVAITELDIAGASAADYEAVTQACLDLENCVGITVWG               293 RMY20991.1           ASGFGSASGVQSALQALA-
GAADEVAITELDIAGASPDAYVTVANACLNVPKCVGITSWG               280 OLN81304.1           AGA---
GSKVLGALQTLAGSGVKEIAITELDIAGAAANDYTAVVNACLQVKQCVGITVWG         275 BAA92882.1           AGG---
SSGVKGALNILAASGVSEVAITELDIAGASSNDYVNVVKACLEVSKCVGITVWG         270 Q96VB6.1           AGG---
SSGVKGALNTLAASGVSEVAITELDIAGASSNDYVNVVEACLEVSKCVGITVWG         270 KAF4307014.1           AGG---
```

```
SSGTTAAMQALC-AAVSECAITELDIAGAAASDYVAVTNACLDIDNCVGITVWG        273 KAH7045322.1          AGG---
ASGTGAAMKALC-AAASECAITELDIAGAAASDYVTATQACLDVENCVGITVWG        273
                 .   . .  *:. :. . .  * *:**:** .*    :  ...***:  :***** **


KAH8651972.1          VRDADSWRTGANPLLFDNSYQPKAAYYAIANALS--                 311
KAH7142868.1          VRDADSWRTGANPLLFDNNYQPKAAYSAIVNALS--                 311
CRG90418.1            TSDAHSWRSSEHPLLFDDNYQPKDAYNAIVTLLS--                 306
KFY28480.1            TSDAHSWRSDEFPLLFDGDYQPKEAYNAIVELLSAA                 309
XP_007712311.1        VRDSDSWRPQGNPLLFDNSYNPKQAYTTVLNALK--                 315
XP_007697074.1        VKDSDSWRPQGNPLLFDNSFNPKQAYTTVLNALK--                 315
XP_007687276.1        VKDSDSWRPQGNPLLFDNSFNPKQAYTTVLNALK--                 315
XynA                  VSDSQSWRPNENPLLFDGSFQPKEAYTGLTTLLA--                 327
RMY20991.1            VSDKDSWRSSDNPLLFDRNYQAKQAYNSVMSALR--                 314
OLN81304.1            VRDPDSWRSQNSPLLFDANFSPKAAYNAIVKALQ--                 309
BAA92882.1            VSDKNSWRSAESPLLFDGNYQPKTAYNAILNAL---                 303
Q96VB6.1              VSDKNSWRSAESPLLFDGNYQPKSAYNAILNAL---                 303
KAF4307014.1          VSDASSWRADSTPLLFDSSYQPKEAYTAIINVL---                 306
KAH7045322.1          VSDANSWRASSSPLLFDASYQPKAAYTAIINAL---                 306
                      .*  ***    ***** .:. * **  :   *
```

Figure S1. Multiple sequence alignment of GH10 endoxylanases. Sequences with similarity to XynA were obtained by BLASTP search. Signal peptides of the sequences, determined by SignalP (https://services.healthtech.dtu.dk/service.php?SignalP-5.0), were removed before alignment. Alignment was carried out with Clustal Omega (https://www.ebi.ac.uk/Tools/msa/clustalo/), using default parameters. Note the lack of sequence conservation of the IDR of XynA (highlighted in red) compared to the other sequences. In the alignment, GenBank accession numbers of the sequences used are indicated, which belong to *Ilyonectria robusta* (KAH8651972.1), *Dactylonectria estremocensis* (KAH7142868.1), *Talaromyces islandicus* (CRG90418.1), *Pseudogymnoascus* sp. (KFY28480.1), *Bipolaris zeicola* (XP_007712311.1), *Bipolaris sorokiniana* (XP_007697074.1), *Bipolaris oryzae* (XP_007687276.1), *Hortaea werneckii* (RMY20991.1), *Colletotrichum chlorophyti* (OLN81304.1), *Aspergillus sojae* (BAA92882.1), *Aspergillus oryzae* (Q96VB6.1), *Botryosphaeria dothidea* (KAF4307014.1) and *Macrophomina phaseolina* (KAH7045322.1).

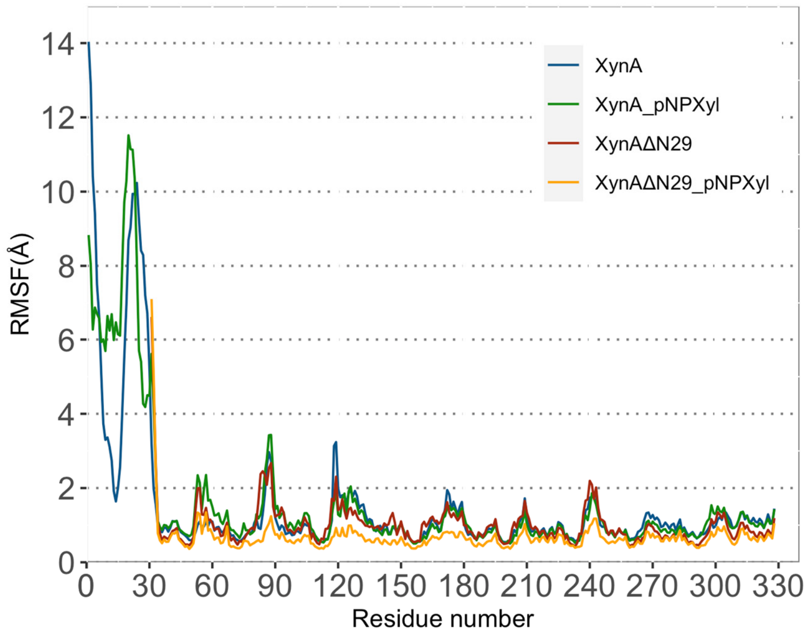

Figure S2. Root mean square fluctuation (RMSF) plot of Cα atoms during simulations. Results for XynA (blue), XynA+pNPXyl (green), XynAΔN29 (red) and XynAΔN29+pNPXyl (yellow) are shown. The X-axis indicates residue number, and the Y-axis represents RMSF value in Å. As expected, the IDR (amino acids 1-29) in XynA and XynA+pNPXyl systems show high fluctuation as compared to other regions of the protein. Position 90 and 120 show peaks at ~3.8 Å, but this is expected because these regions are loops, which are often highly mobile even in stable proteins. Relevant fluctuations in other regions of interest (for example, catalytic amino acids E160 and E263) were not observed.

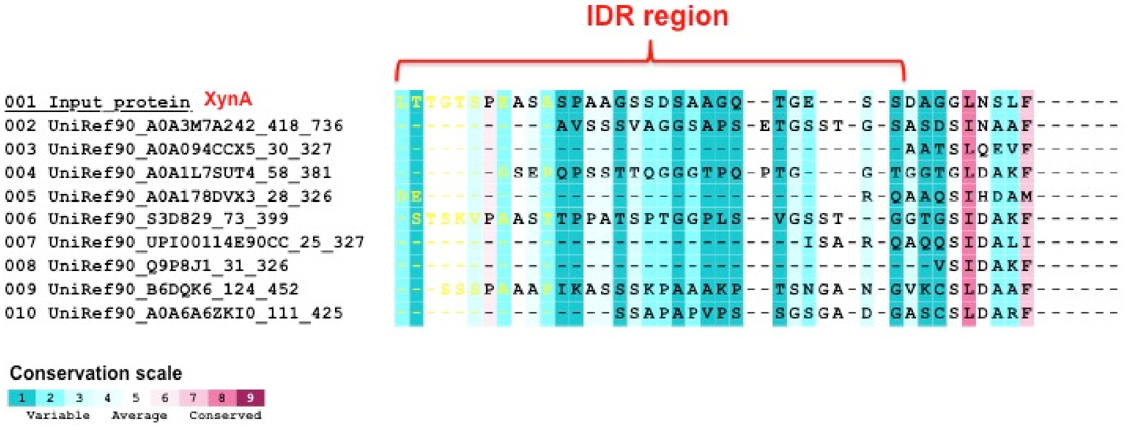

Figure S3. ConSurf analysis of evolutionary conservation of XynA. The analysis included 149 proteins similar to XynA. Only the amino end region of XynA and the first 10 sequences are shown. Amino acids were ranked on a conservation scale as follows: levels 1 to 3 are variable, levels 4 to 6 are average, and levels 7 to 9 are conserved. For amino acids highlighted in yellow, ConSurf does not have sufficient data for the calculation of the degree of conservation.

Table S1. Percentage of secondary structure content of XynA and XynAΔ29. Values were obtained from the analysis of CD spectra using BeStSel.

Enzyme	α-helix	antiparallel β-sheet	parallel β-sheet	turn	others[1]
XynA	10.7	20.5	14.4	11.3	43.1
XynAΔ29	5.2	20.8	7.8	12.8	53.4

[1] The category "others" includes short secondary structures (3_{10} helix, π-helix, β-bridge) and irregular structures. For further details see reference [47] cited in the main text.

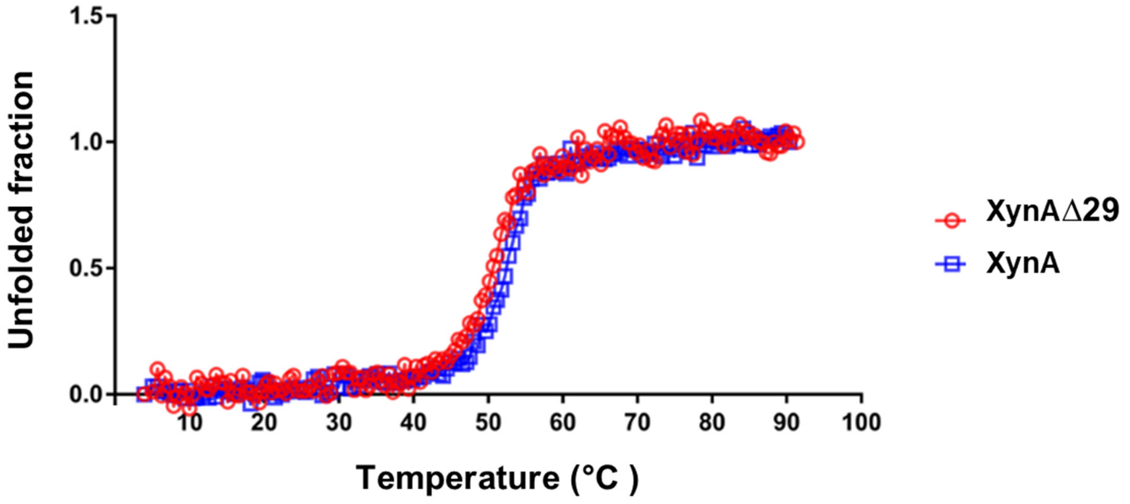

Figure S4. Thermal unfolding of XynA and XynAΔ29. Spectra were recorded by circular dichroism following the molar ellipticity at 220 nm. The melting temperatures determined were 52 °C for XynA and 50 °C for XynAΔ29.

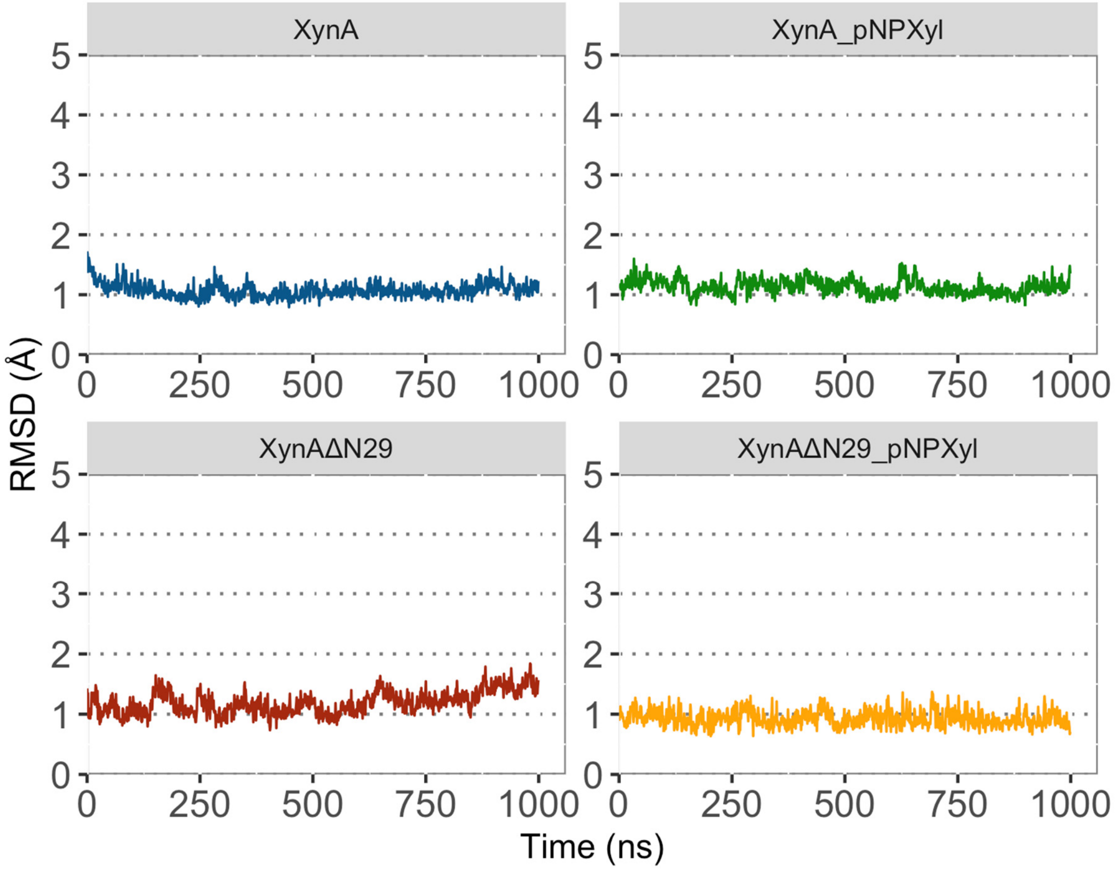

Figure S5. Root mean square deviation (RMSD) of backbone atoms as a function of time. As described in Materials and methods, the systems were stabilized at 40 ns. After that, 1-microsecond trajectories were analyzed which are shown in this figure. Upper panels: XynA (blue) and XynA+pNPXyl (green). Lower panels: XynAΔN29 (red) and XynAΔN29+pNPXyl (yellow).
